# The transition of adult patients with childhood-onset chronic diseases from pediatric to adult healthcare systems: a survey of the perceptions of Japanese pediatricians and child health nurses

**DOI:** 10.1186/1751-0759-6-8

**Published:** 2012-03-20

**Authors:** Yuko Ishizaki, Mitsue Maru, Hirohiko Higashino, Shoko Katsumoto, Kyoko Egawa, Yoshitoki Yanagimoto, Teruyo Nagahama

**Affiliations:** 1 Research Group on Promotion of Transition of Care from Pediatric to Adult Health Services for Young Adults with Childhood-onset Chronic Disease; 2 Department of Pediatrics, Kansai Medical University, Moriguchi, Osaka, Japan; 3 Division of International Nursing Development, Tokyo Medical and Dental University, Bunkyo, Tokyo, Japan; 4 Higashino Clinic, Higashi-Osaka, Osaka, Japan; 5 Graduate School of Human Life Science, Osaka City University, Osaka, Japan; 6 Department of Pediatrics, Kansai Medical University, Fumizonocho 10-15, Moriguchi, Osaka 570-8506, Japan

**Keywords:** Transition of care, Pediatrician, Child health nurse, Adult patients with child-onset chronic illness, Psychosomatic problems

## Abstract

**Background:**

Advances in medical science have enabled many children with chronic diseases to survive to adulthood. The transition of adult patients with childhood-onset chronic diseases from pediatric to adult healthcare systems has received attention in Europe and the United States. We conducted a questionnaire survey among 41 pediatricians at pediatric hospitals and 24 nurses specializing in adolescent care to compare the perception of transition of care from pediatric to adult healthcare services for such patients.

**Findings:**

Three-fourths of the pediatricians and all of the nurses reported that transition programs were necessary. A higher proportion of the nurses realized the necessity of transition and had already developed such programs. Both pediatricians and nurses reported that a network covering the transition from pediatric to adult healthcare services has not been established to date.

**Conclusions:**

It has been suggested that spreading the importance of a transition program among pediatricians and developing a pediatric-adult healthcare network would contribute to the biopsychosocial well-being of adult patients with childhood-onset chronic disease.

## Background

Advances in medicine have dramatically improved the prognosis for children with chronic diseases that were previously fatal in childhood and have allowed them to survive to adulthood [1-3]. In Japan, 1,000 patients with childhood-onset chronic disease (CCD) attain adulthood every year, and many of them do not have serious sequelae or disabilities [4]. These high survival rates have increased the number of adolescents confronting the issue of transition from pediatric to adult healthcare [2,3,5-11]. In addition, the prevalence of some kinds of chronic illnesses in childhood is increasing [5]. These adolescent patients are still developing socially, and they often lack social experiences because of their childhood disease and have difficulties in adapting to both adult social life within their community and adult healthcare systems [12]. Therefore, programs are required to ensure a seamless transition of medical care in childhood and adolescence to that in adulthood and to help children grow socially and become independent, working adults.

The transition of young people with CCD and disability from pediatric to adult healthcare has recently received significant attention in literature. Transition has been defined as a multi-faced, active process that attends to the medical, psychosocial, and educational needs of adolescents as they move from child to adult centered care [5]. In Europe, the United States, and Australia, the importance of healthcare during this transition period has been acknowledged; there are guidelines for transition care and many studies have reported its effectiveness [13-15]. The transition program is mostly problem oriented and its participants include patients, families, pediatricians, nurses, adult healthcare providers, and other healthcare professionals [13-16]. Discussions have begun among medical professionals in Japan on whether pediatric or adult healthcare services should provide care to adult patients with CCD [17].

Since implementation of this transition requires the cooperation of medical professionals (i.e., mainly pediatricians and nurses), it is important to ascertain the views of these professionals on this issue. To date, no survey has investigated the policies of different types of healthcare professionals on transition care for pediatric patients with chronic disease attaining adulthood in Japan. We conducted a questionnaire survey to compare awareness with regard to the problems involved in healthcare for adults with CCD and the transition programs between pediatricians at pediatric hospitals and nurses specializing in adolescent care.

## Methods

The subjects comprised 41 pediatricians and 24 nurses working in pediatric units. The pediatricians were managers or department heads of 23 Japanese Association of Children's Hospitals and Related Institutions. The nurses were all head nurses of children's hospitals attending a continuing education program on the subject of transition sponsored by the Japanese Association of Adolescent Nursing. The protocol of this study was approved by the Ethical Committee of the Japanese Association of Children's Hospitals and Related Institutions and the Japanese Association of Adolescent Nursing.

The length of work experience of the respondents was 27.1 ± 6.6 years (range, 12-40 years) for pediatricians and 11.4 ± 9.4 years (range, 2-30 years) for nurses. Thirty-eight pediatricians were male (92.7%) and all the nurses were female. Subspecialties within pediatrics of the participating pediatricians included neurology (N = 6), nephrology (N = 6), cardiovascular disease (N = 4), allergy/collagen disease/infectious disease (N = 4), and others.

The questionnaire included questions on background characteristics, adult patients with CCD in everyday practice, awareness of transition from pediatric to adult healthcare, factors determining the patients' transition from pediatric to adult healthcare, and the handling of the psychosocial problems of adult patients. The questionnaires, with a cover letter detailing the purpose of the study and explaining that participation is voluntary, were distributed by the controller to pediatricians or by the secretariat of the continuing education program to nurses. Completed questionnaires were returned to the controller anonymously in a sealed and stamped envelope. The chi-square test was performed to compare the responses of the pediatricians and nurses. A p value of .05 or less was considered to be statistically significant. The statistical software employed in this analysis was the SPSS 11.0J [18].

## Results

With regard to the management of adult patients with childhood-onset disease, 12.2% of the pediatricians reported that the patients had received outpatient care only and 75.6% of the pediatricians and all of the nurses reported that the patients had received both outpatient and inpatient care. In total, 34.1% of the pediatricians and 25.0% of the nurses reported that they were capable of managing the psychosocial problems of adult patients.

Seventy-eight percent of the pediatricians and all of the nurses reported that a transition program was necessary. When asked whether they would transfer adult patients with CCD to adult healthcare services, 63.4% of the pediatricians and 91.7% of the nurses answered in the affirmative. The pediatricians answered this question with either "no" or "undecided," primarily because they did not find experts in adult healthcare services. Among the respondents, 25.8% of the pediatricians and 16.7% of the nurses reported that a network of adult healthcare services was available for such patients. With regard to transition processes, 22.0% of the pediatricians and 37.5% of the nurses reported that an established process for instruction on patient self-care was already in place. The responses "a transition program is necessary," "transfer adult patients with childhood-onset disease to adult healthcare services," and "the process of transition has been determined" were given significantly more often by the nurses than the pediatricians.

More than 70% of all the respondents indicated the patient's age and social factors (e.g., starting school, employment, or change of residence) and more than 50% of them indicated the patient's psychosocial maturity to be factors involved in the transition (Table 1). As determining factors, the pediatricians mentioned "unmanageable problems in adult patients" more frequently, but "age of patients," "social factors," and "withdrawal of medical funding" less frequently than the nurses.

**Table 1:** Factors determining the transition of patients from pediatric to adult healthcare

	Pediatricians	Nurses
	(N = 41)	(N = 24)
Age of patients**	22 (53.0%)	18 (75.0%)
Psychosocial development of patients	16 (39.0%)	14 (58.3%)
Social factors**	25 (61.0%)	19 (79.2%)
Stability of disease condition	12 (29.3%)	11 (45.8%)
Withdrawal of medical funding**	0	4 (16.7%)
Unmanageable problems occurring in adult patients*	12 (29.3%)	2 (8.3%)

(The chi-square test: *, p < 0.01; **, p < 0.001)

## Discussion

We investigated the views of pediatricians and nurses on transition of care for pediatric patients with chronic disease who are about to become adults. Whereas the sample size was small, the targets of the present survey were pediatricians and nurses who were department heads at Japan's leading institutions for child health and development, and as a preliminary report, the opinions of the respondents possibly reflect the principles and practical conditions applying to transition of care in pediatric and adolescent healthcare in Japan.

The survey results showed that three-fourths of the pediatricians and all of the nurses surveyed believed that transition programs are necessary. However, only 22% of the pediatricians and 37.5% of the nurses had established processes in place. Compared with the pediatricians in this study, a higher proportion of the nurses realized the necessity of transition from pediatric to adult healthcare services and had already developed and implemented such programs. Although manuals on transition programs have been published by both physicians and nurses in Europe and the United States, the first manual in Japan was produced by nurses as recently as 2010 [19]. According to the findings of our research, spreading the importance of this issue among pediatricians is necessary for a smooth transition of care.

Both the pediatricians and nurses attached importance to the patient's age, psychological development, and social factors when determining the transition of care. To an open-ended question as to the reason why pediatricians cannot transfer patients to adult healthcare services, many pediatricians reported a shortage of experts in adult healthcare services, and both pediatricians and nurses pointed out the lack of an established professional network covering the transition from pediatric to adult healthcare services. These findings are similar to previous reports from other countries, which mentioned the following barriers to transition of healthcare systems: lack of recognition of transition, sporadic collaboration between specialists in pediatric and adult healthcare, and shortage of training in managing CCD among adult caregivers [2,16].

Only one-third of the pediatricians and one-fourth of the nurses stated that they were able to handle the psychosocial problems of adult patients. This suggests that healthcare professionals in pediatric departments do not appropriately address the psychosocial problems of adult patients with CCD. On the other hand, the prognosis is generally not good, although specialists in psychosomatic medicine do their best when dealing with such problems. Psychosocial problems need to be prevented, and therefore, it is necessary to establish a transition program to improve psychosocial health.

In conclusion, it is important to disseminate knowledge of transition programs among pediatricians and to develop a pediatric-adult healthcare network by communication between healthcare professionals in pediatric and adult healthcare services for the biopsychosocial well-being of adult patients with CCD.

## Abbreviations

CCD: Childhood-onset chronic disease.

## Competing interests

The authors declare that they have no competing interests.

## Authors' contributions

YI, MM and HH participated in the design of this study. YI and YY drafted the manuscript. MM is a grant holder. SK and KE performed statistical analysis. TN supervised the manuscript. All authors read and approved the final version of this manuscript.
